# Ectopic Ureteral Insertion into the Seminal Vesicle Causing Recurrent Epididymitis in a 24-Year-Old

**DOI:** 10.1089/cren.2015.29006.bos

**Published:** 2015-10-01

**Authors:** Timothy Charles Boswell, Tracy Marien, Stanley Duke Herrell

**Affiliations:** ^1^ College of Medicine, University of Tennessee Health Science Center, Memphis, Tennessee.; ^2^ Department of Urologic Surgery, Vanderbilt University Medical Center, Nashville, Tennessee.

## Abstract

A 24-year-old male was found to have recurrent epididymitis secondary to ectopic ureteral insertion to the seminal vesicle. His ipsilateral kidney was atrophic and ectopic in the pelvis, suggesting a complex failure of embryological development. He was successfully treated with robot-assisted laparoscopic nephroureterectomy.

## Clinical History

A 24-year-old male with intermittent episodes of right epididymitis over several months presented to his local urologist with increasing scrotal pain and swelling. This episode of epididymitis had been treated by his primary physician with a course of doxycycline followed by an additional course of fluoroquinolone for persistent symptoms. The patient denied nausea, vomiting, abdominal pain, back pain, other urinary symptoms, gross hematuria, urethral discharge, history of sexually transmitted disease, or recent scrotal trauma. He specifically denied hematospermia or pain with ejaculation. He reported a similar episode and symptoms ∼3 years ago which had resolved spontaneously. He denied any other urologic personal or family history. The patient had no other pertinent medical, genitourinary, or sexual history.

## Physical Examination

The patient was a young well-developed male. He was afebrile with normal vital signs. On genitourinary examination, the right vas and epididymis were tender, firm, and indurated on palpation.

## Diagnosis

Urinalysis was negative for nitrites, leukocyte esterase, or blood. Scrotal ultrasound (US) was remarkable for an enlarged right epididymis with hyperemia. Given the recurrent episodes of epididymitis, further workup was performed.

**FIG. 1.**
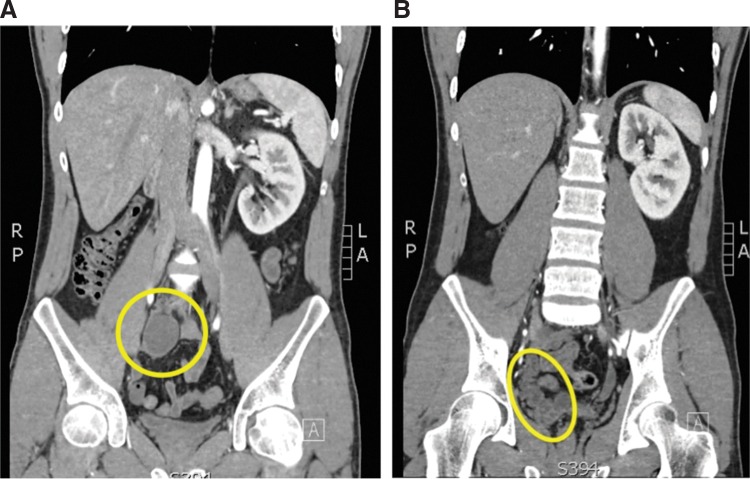
Atrophic and ectopic right kidney. **(A)** A coronal cut of the CT scan showing the atrophic right pelvic kidney (*yellow circle*). **(B)** On this coronal cut of the CT scan, the ureter and seminal vesicle complex can be seen (*yellow ellipse*).

Abdominal US demonstrated a normal left kidney and bladder with the absence of the right kidney in the retroperitoneum. A CT scan of the abdomen and pelvis was obtained and revealed a cystic mass in the right pelvis consistent with a possible hydronephrotic, atrophic right pelvic kidney. A tubular structure suspicious for the ureter appeared to be inserting into a cystic right seminal vesicle (Fig. 1). The left kidney showed evidence of appropriate compensatory hypertrophy. No clear identifiable vascular supply to this presumed atrophic kidney was noted, although it lay in close proximity to the right internal and external iliac vessels. The pelvic kidney had no definable cortical enhancement, and delayed images demonstrated no excretion of contrast into the collecting system. Serum creatinine was 1.15 mg/dL.

Ectopic kidney with an ectopic ureteral insertion to the ipsilateral seminal vesicle was suspected as the cause of the patient's recurrent epididymitis. In men, ectopic ureteral implantation into the seminal system (seminal vesicle, ejaculatory duct, vas deferens, or epididymis) tends to present with peak incidence in the third decade of life through symptoms associated with voiding, ejaculation, or pain of the perineum or genitals. In contrast, patients with ectopic ureteral insertion into the urinary tract (bladder neck and posterior urethra) tend to present younger (<15 years old) and with urinary-tract infections or trunk pain.^1^ Ectopic ureter is also frequently associated with dysgenesis of the ipsilateral kidney, especially in the setting of a single-system ectopic ureter as in our case. An embryological failure of the mesonephric or Wolffian duct explains the connection between ectopic ureter and concurrent ipsilateral renal malformation.

Zinner syndrome represents a specific entity along the spectrum of ureteral malformation and renal maldevelopment in males, which has a potential relevance to our case. It is defined as the triad of unilateral renal agenesis, ipsilateral seminal vesicle cyst, and ejaculatory duct obstruction. Furthermore, ureteral absence or ectopia commonly occur as associated findings in this syndrome. The presentation of Zinner syndrome typically revolves around the mass effect from the cystic seminal vesicle: urinary tract infection, epididymitis, perineal pain, dysuria, hematuria, or infertility. Frequently, patients are asymptomatic and the anomaly is discovered incidentally on imaging. Symptomatic presentation occurs most commonly in young adulthood. In a patient with suggestive presentation and the discovery of renal agenesis, viable options for targeted evaluation of Zinner syndrome include digital rectal examination (to assess for supraprostatic mass), transrectal US, CT, MRI, intravenous urography, and cystourethroscopy.^2^ While the patient in our case did not have complete renal agenesis but rather severe renal atrophy, all other aspects of this case are similar to Zinner syndrome. Case reports of Zinner syndrome do exist that describe a renal remnant,^3^ suggesting that some experts would classify this as a case of Zinner syndrome. Conservative management with fertility workup is recommended for asymptomatic to mild cases; more severe cases typically require surgical treatment, for which laparoscopic and robotic approaches are becoming increasingly favored.^4^

## Intervention

The patient was referred to our center for surgical management and counseled that his recent bouts of recurrent epididymitis were likely secondary to an atrophic right pelvic kidney with ectopic ureteral insertion into the ipsilateral seminal vesicle. He elected to undergo right nephroureterectomy, and a minimally invasive approach was recommended to decrease recovery time. A robotic approach was selected by the surgical team. Informed consent was obtained.

**FIG. 2.**
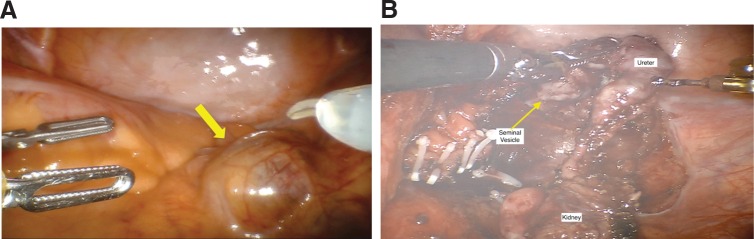
Intraoperative view of right pelvic kidney. **(A)** A view of the pelvic kidney (*yellow arrow*) before dissection. **(B)** Shows the ureter inserting into the right seminal vesicle.

**FIG. 3.**
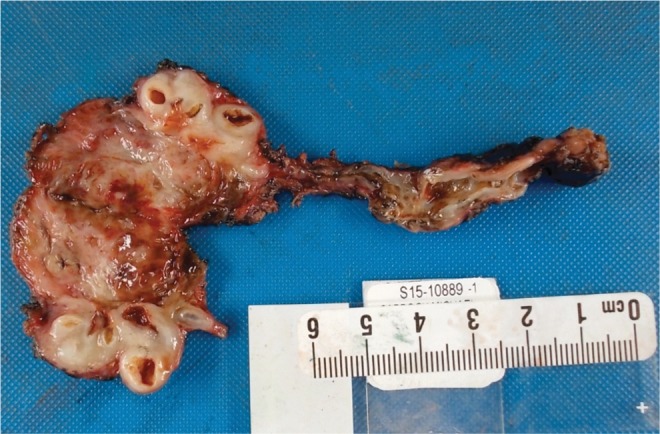
Final pathology: atrophic kidney and dilated ureter.

After general anesthesia was administered, the patient was placed in the standard low lithotomy position for robotic pelvic surgery. Cystoscopy confirmed an orthotopic left ureteral orifice, the absence of a right ureteral orifice, and an extrinsic mass posterior to the right hemitrigone area. Port placement was similar in configuration to robotic prostatectomy, although slightly more cephalad. The cystic mass (right kidney) was identified in the pelvis (Fig. 2A). With careful dissection and manipulation, the kidney was freed from its attachments. Due to limited space and visualization angles, the kidney was decompressed. Structures consistent with atrophic renal vasculature between the kidney and external iliac vessels were carefully dissected and ligated with multiple clips. The cystic and redundant ureter was dissected down to the right seminal vesicle where its attachment to the seminal vesicle tip on the right was transected (Fig. 2B). The seminal vesicle tip was carefully oversewn with absorbable suture. Estimated blood loss was 25 mL, and the operative time was 164 minutes. The patient tolerated the procedure well and was discharged home on the second postoperative day. Final pathology showed a benign, hydronephrotic, atrophic kidney and seminal vesical cysts (Fig. 3).

## Follow-Up

During follow-up, the patient had complete symptom relief, no recurrences of epididymitis, and significant resolution of the vasal and epididymal induration initially present on examination.

## Outcomes

While anatomic abnormalities of the genitourinary tract are rarely diagnosed in adulthood, a history of recurrent epididymitis in a young man should prompt evaluation with imaging studies to assess for a potentially correctable pathology. Minimally invasive surgical techniques, such as robotics, can facilitate the safe and effective management of uncommon anatomic pathology.

## Abbreviations Used

CT: computed tomography
MRI: magnetic resonance imaging
US: ultrasound
